# Probiotic Potential of *Limosilactobacillus reuteri* JS-51 from Traditional Fermented Jiangshui: Screening, Characterization, and Hypocholesterolemic Effects

**DOI:** 10.3390/foods15193554

**Published:** 2026-10-06

**Authors:** Xiaohui Tan, Xin Zhang, Longxiang Wang, Yaping Zhang, Zhiwei Shen, Haokun Liu, Hao Wang, Fuyun Zhang

**Affiliations:** 1 Dalian Ocean University School of Food Science and Engineering, Dalian 116023, China; xxdooollar@gmail.com (X.T.); zhangxin20260818@163.com (X.Z.);; 2 Technology Centre of Dalian Customs District, Dalian 116001, China

**Keywords:** *Limosilactobacillus reuteri* JS-51, *Jiangshui*, hypocholesterolemic effect, screening, probiotic characterization

## Abstract

Hypercholesterolemia is a major risk factor for cardiovascular and hepatic diseases. Recently, probiotics have garnered significant attention as a promising, non-pharmacological approach to regulate lipid metabolism. In this study, we screened potential cholesterol-lowering lactic acid bacteria from *Jiangshui*, a traditional Chinese fermented vegetable beverage. Initially, 52 acid-producing isolates were obtained using CaCO_3_-supplemented MRS agar. Subsequent screening for bile salt hydrolase (BSH) activity yielded five promising strains. These isolates were further evaluated for gastrointestinal tolerance, cell surface hydrophobicity, and in vitro cholesterol removal capacity. Strain JS-51, identified as *Limosilactobacillus reuteri* via 16S rRNA sequencing, exhibited superior probiotic properties. It demonstrated high cell surface hydrophobicity (72.36 ± 1.24%), robust survival under harsh conditions (pH 3.0 and 0.5% bile salts), and the highest in vitro cholesterol removal rate (55.15 ± 1.87% at 48 h). The in vivo hypocholesterolemic efficacy of *L. reuteri* JS-51 was then evaluated in a high-fat diet-induced Sprague Dawley rat model. Following a four-week daily oral administration at doses of 10^6^, 10^7^, and 10^8^ CFU/mL, JS-51 elicited a dose-dependent reduction in serum total cholesterol, triglycerides, and low-density lipoprotein cholesterol (*p* < 0.001), alongside an increase in high-density lipoprotein cholesterol. Histopathological analysis revealed observable morphological amelioration of hepatic steatosis, a reduction in the liver index by up to 5.56%, and the restoration of normal hepatocyte architecture. These phenotypic findings indicate that *L. reuteri* JS-51 possesses robust in vitro probiotic potential and effective in vivo lipid-lowering capabilities, highlighting its potential as a functional starter culture or dietary supplement for managing hypercholesterolemia.

## 1. Introduction

Hypercholesterolemia is a major risk factor for atherosclerotic cardiovascular disease (ASCVD) [1,2]. Although statins and other pharmacotherapies can effectively reduce lipid levels, their potential side effects and issues regarding long-term patient adherence [3] highlight the urgent need for safer, non-pharmacological alternatives, such as functional foods and dietary probiotics [4].

Extensive in vitro and in vivo evidence supports the hypocholesterolemic potential of specific probiotics. Generally proposed pathways include the enzymatic deconjugation of bile acids by bile salt hydrolase (BSH), which limits lipid absorption and promotes *de novo* bile acid synthesis from systemic cholesterol in the liver. Furthermore, direct assimilation or co-precipitation of cholesterol with deconjugated bile salts, the incorporation of cholesterol into bacterial cell membranes [5], and the modulation of gut microbiota composition to favor lipid-lowering taxa are also well-documented modes of action [6,7]. Clinical meta-analyses have demonstrated that certain probiotic strains, notably within the genera *Lactobacillus* (reclassified as *Lactiplantibacillus*, etc.) and *Bifidobacterium*, can reduce serum total cholesterol (TC) by 4–12% [8] and low-density lipoprotein cholesterol (LDL-C) by 5–15% in hypercholesterolemic individuals [8,9], exhibiting efficacy comparable to moderate dietary interventions. Among these, *Limosilactobacillus reuteri* (formerly *Lactobacillus reuteri*) strains have shown particular promise [10], with clinical trials reporting significant reductions in TC, LDL-C, and apolipoprotein B-100, alongside improvements in inflammatory markers.

Traditional fermented foods represent a rich reservoir of beneficial microorganisms shaped by centuries of natural selection in diverse ecological niches [11,12,13]. In China, spontaneously fermented vegetables such as *suancai*, *paocai*, and *Jiangshui* harbor complex microbial communities dominated by lactic acid bacteria (LAB). *Jiangshui*, a traditional vegetable beverage prevalent in northwest China (particularly Ningxia, Gansu, and Shanxi provinces) [14,15], is prepared from celery, cabbage, or other leafy vegetables through natural lactic acid fermentation without added salt or commercial starters. This process yields a sour, refreshing product consumed as a soup base or beverage, valued for its digestive and health-promoting properties in local folk medicine [16,17]. High-throughput sequencing studies have revealed that the *Jiangshui* microbiota is primarily composed of Firmicutes (predominantly *Lactobacillus* and *Pediococcus*), with minor contributions from Proteobacteria, yeasts, and molds [18,19]. These dominant LAB not only contribute to flavor development but also exhibit functional attributes, including antioxidant activity, pathogen inhibition, and potential lipid-regulating effects [20]. Despite its cultural significance and microbial richness, *Jiangshui* remains underexplored as a source of novel functional strains compared to more extensively studied ferments like kimchi or yogurt [21].

The growing demand for ethnic and traditional fermented foods highlights their potential as promising sources of novel bio-functional candidate probiotic strains. Unlike commercial strains often derived from mild dairy matrices, LAB from *Jiangshui* have undergone evolutionary selection within a uniquely harsh and acidic ecological niche (pH 3.2–3.5). We hypothesized that this rigorous acidic domestication confers superior gastrointestinal survival capabilities to indigenous LAB. *L. reuteri*, which is frequently isolated from such traditional ferments, naturally demonstrates strong BSH activity, high cell surface hydrophobicity (facilitating intestinal adhesion), and a robust cholesterol assimilation capacity. These integrated phenotypic traits are essential for strains to exert effective hypocholesterolemic actions in vivo [22].

Therefore, this study aimed to systematically screen and characterize potential cholesterol-lowering LAB from traditional Ningxia *Jiangshui*. The primary focus was placed on isolating strains that exhibit strong BSH activity, robust gastrointestinal tolerance, and high in vitro cholesterol removal efficiency. The most promising isolate, subsequently identified as *L. reuteri* JS-51, was further evaluated in a high-fat/high-cholesterol diet-induced hypercholesterolemic rat model. By assessing its dose-dependent phenotypic effects on serum lipid profiles, hepatic steatosis, and overall liver architecture, this work seeks to provide objective evidence for the valorization of Chinese ethnic fermented foods as valuable sources of functional candidate probiotic strains for cardiovascular health management.

## 2. Materials and Methods

### 2.1. Reagents and Materials

De Man, Rogosa and Sharpe (MRS) broth was purchased from Hangzhou Baisi Biotechnology Co., Ltd. (Hangzhou, China). Calcium carbonate (CaCO_3_) and the Gram staining kit were obtained from Beijing Solarbio Science & Technology Co., Ltd. (Beijing, China) and Changde BKMAM Biotechnology Co., Ltd. (Changde, China), respectively. Sodium chloride was supplied by Tianjin Damao Chemical Reagent Factory (Tianjin, China). Cholesterol powder and xylene were purchased from Sinopharm Chemical Reagent Co., Ltd. (Shanghai, China). *o*-phthalaldehyde (OPA) was obtained from Shanghai Yuanye Bio-Technology Co., Ltd. (Shanghai, China), while glacial acetic acid and chloroform were supplied by Tianjin Fuyu Fine Chemical Co., Ltd. (Tianjin, China). Other general chemicals were of analytical grade. Bile salts (porcine bile extract), water-soluble cholesterol, and cholesterol standards were acquired from Sigma-Aldrich (St. Louis, MO, USA). For strain identification, the bacterial genomic DNA extraction kit was obtained from Shanghai Jerry Bioengineering Co., Ltd. (Shanghai, China), and PCR primers were synthesized by Sangon Biotech (Shanghai, China). In the animal study, male Sprague–Dawley rats and experimental diets (basal and high-fat) were supplied by SPF (Suzhou) Biotechnology Co., Ltd. (Suzhou, China). The high-fat diet was formulated with 1% cholesterol and 20.2% egg yolk powder. Diagnostic kits for the quantitative determination of serum total cholesterol (TC), triglycerides (TGs), high-density lipoprotein cholesterol (HDL-C), and low-density lipoprotein cholesterol (LDL-C) were purchased from Mindray Bio-Medical Electronics Co., Ltd. (Shenzhen, China).

### 2.2. Screening, Isolation, and Purification of Cholesterol-Degrading Bacterial Strains from Jiangshui

Traditional *Jiangshui* from Ningxia, China, naturally fermented for 7 days at room temperature, was used in this study. To ensure consistency, the same batch of *Jiangshui* was utilized throughout the entire experiment and stored at 4 °C. Under a laminar flow hood, the *Jiangshui* was transferred into 10 mL sterile centrifuge tubes. After vortex mixing, *Jiangshui* was serially diluted with sterile saline to concentrations of 10^−1^, 10^−2^, and 10^−3^. The diluted *Jiangshui* samples were evenly distributed on the surface of MRS CaCO_3_ solid medium containing 5 g/L CaCO_3_ by the spread plate method. The etri dishes were placed upside down in an anaerobic incubator at 37 °C for 36 h. The colonies with transparent halos around them were selected to be inoculated onto MRS solid medium by the streak plate method and were incubated in an anaerobic incubator at 37 °C for 24–48 h. This step was repeated until a pure culture was obtained. Subsequently, bile salt hydrolase (BSH) solid medium and the filter paper disc method were used to screen the strains with cholesterol removal potential by observing whether there was a precipitation ring around them [22,23].

### 2.3. Identification of Cholesterol-Degrading Bacterial Strains from Jiangshui

The isolated strains were identified by Gram staining, morphological observation, and 16S rRNA gene sequencing. After incubation in MRS broth at 37 °C for 18–24 h, the isolates were streaked onto fresh MRS agar to obtain single colonies, and representative colonies were Gram-stained using a commercial kit according to the manufacturer’s instructions. Bacterial cells were suspended in sterile physiological saline, heat-fixed on glass slides, and sequentially stained with crystal violet, iodine solution, alcohol, and safranin before being examined with a light microscope. Genomic DNA of the strains was extracted using a bacterial genomic DNA extraction kit (Shanghai Jerry Bioengineering Co., Ltd., Shanghai, China). The 16S rRNA gene was amplified with universal primers 27F (5′–AGAGTTTGATCCTGGCTCAG–3′) and 1492R (5′–GGTTACCTTGTTACGACTT–3′) [15]; purified PCR products were sequenced bidirectionally by Sangon Biotech (Shanghai, China). The sequences of 16S rDNA were compared with reference sequences in the National Center for Biotechnology Information (NCBI) database using the online BLASTn search tool (https://blast.ncbi.nlm.nih.gov/Blast.cgi), aligned with ClustalW, and analyzed phylogenetically with MEGA 11 software [24] by the neighbor-joining method with 1000 bootstrap replications.

### 2.4. Determination of Biological Performance of Cholesterol-Degrading Bacterial Strains

#### 2.4.1. Growth Ability of Strains in Different pH Environments

The activated bacterial cells were harvested by centrifugation at 1500× *g* for 10 min at 4 °C, washed twice with sterile phosphate-buffered saline (PBS, pH 7.2), and resuspended to approximately 10^8^ CFU/mL. The pH of MRS broth was adjusted to 2, 3, 4, 5, 6, 7, 8, 9, 10, 11, and 12 using 4 M HCl or 4 M NaOH. The cell suspensions (10 µL) were inoculated into MRS broth (10 mL) adjusted to different pH values and incubated at 37 °C. Optical density at 600 nm (OD_600_) was measured at 0, 4, 8, 12, 16, and 24 h to evaluate the growth of isolated strains in different pH conditions.

#### 2.4.2. Acid Tolerance of Strains

The acid tolerance of the strains was evaluated by detecting their survival rate in MRS broth at pH 2 and 3. The bacterial cells were resuspended in MRS broth adjusted to pH 2 and 3, respectively. Suspensions (10^8^ CFU/mL) were incubated at 37 °C for 2 h or 4 h. Then viable cells were enumerated using the plate count method. The survival rate was calculated according to the following equation:

$$\mathrm{Survival rate}(\%)=\frac{A_{1}}{A_{0}}100\%$$

where A_0_ represents the viable count before incubation, and A_1_ represents the viable count after incubation.

#### 2.4.3. Bile Salt Tolerance of Strains

Cell suspensions (10 µL, 10^8^ CFU/mL) were inoculated into MRS broth (10 mL) containing bile salt at final concentrations of 0.3% (*w*/*v*) or 0.5% (*w*/*v*), and incubated at 37 °C for 0, 2, 4, 6, 8, and 10 h, respectively. The growth of strains was monitored by measuring OD_600_. The tolerance to bile salts was evaluated by comparing growth profiles with control cultures without bile salts.

#### 2.4.4. Hydrophobicity of Bacterial Cell Surface

Hydrophobicity of the cell surface was determined using the microbial adhesion to hydrocarbons (MATH) assay [25,26]. Activated cells were harvested by centrifugation at 1500× *g* for 10 min at 4 °C, washed twice with sterile PBS, and resuspended in sterile saline to an optical density at 600 nm (OD_600_) of 0.60 ± 0.05. The initial absorbance of the cell suspension was recorded as A_0_. Then, 3 mL of the standardized cell suspension was transferred to a test tube, and 1 mL of xylene or chloroform was added. The mixture was vortexed for 3 min and left to stand at room temperature for 20 min to separate the two phases. The aqueous phase was carefully collected, and its OD_600_ was measured and recorded as A_1_. Cell surface hydrophobicity was calculated according to the following equation:

$$\mathrm{Surface hydrophobicity}(\%)=\left(1-\frac{A_{1}}{A_{0}}\right)100\%$$

where A_0_ is the absorbance of the bacterial suspension before mixing with the organic solvent, and A_1_ is the absorbance of the aqueous phase after being mixed and phase separated.

### 2.5. The Cholesterol Removal Ability of Strains In Vitro

The cholesterol removal ability of the isolates in vitro was evaluated using the *o*-phthalaldehyde (OPA) method [22,27], which measures the disappearance of cholesterol from the supernatant. A cholesterol standard curve was first established. Briefly, a series of cholesterol standard solutions (0–0.04 mg/mL) was prepared, mixed with glacial acetic acid, MRS broth, and OPA working solution, and left to stand for 10 min. Then the mixed acid was added and mixed well for color development. After incubation at room temperature for 10 min, the absorbance was measured at 550 nm, and a standard curve was constructed by plotting cholesterol concentration against OD_550_.

For the assay of bacterial cholesterol removal, activated cultures were inoculated (3%, *v*/*v*) into MRS broth and incubated at 37 °C for 18 h. Cells were harvested by centrifugation at 1500× *g* for 10 min at 4 °C, washed twice with 0.85% (*w*/*v*) sterile physiological saline, and resuspended in saline to obtain cell suspensions of approximately 10^8^ CFU/mL. The standardized cell suspensions were inoculated at 3% (*v*/*v*) into 6 mL MRS broth supplemented with cholesterol (0.024 mg/mL) and incubated at 37 °C for 12, 24, and 48 h, respectively. Cultures were centrifuged at 8000× *g* for 10 min, and 0.5 mL of the supernatant was collected and reacted sequentially with glacial acetic acid, OPA working solution, and mixed acid. After standing at room temperature for color development, the absorbance at 550 nm was recorded, and the residual cholesterol concentration in the supernatant was calculated from the standard curve. Cholesterol removal was expressed as a percentage and calculated according to the following equation:

$$\mathrm{Cholesterol removal}\left(\%\right)=\frac{B-A}{B}100\%$$

where B is the cholesterol concentration in the medium before incubation and A is the cholesterol concentration in the supernatant after incubation.

### 2.6. Evaluation of Hypocholesterolemic Efficacy of Strain JS-51 in Rats

#### 2.6.1. Construction of High-Fat Diet (HFD) Induced Rat Model

Seventy-five male Sprague-Dawley (SD) rats (180–200 g) were purchased from SPF (Suzhou) Biotechnology Co., Ltd. (Suzhou, China). The rats were kept in a clean room (temperature 20–24 °C, humidity 50 ± 5%, 12 h light/dark cycle) with free access to water and food for 7 days to adapt to the environment.

Then the rats were randomly divided into two groups: a Normal Control group (15 rats) fed a basal diet, and a Model group (60 rats) fed with a high-fat diet to induce hypercholesterolemia. The composition of the HFD (per 100 g) consisted of 78.8 g basal feed, 1 g cholesterol, and 20.2 g egg yolk powder. After 4 weeks of feeding, blood from non-fasted rats was collected to quantify serum total cholesterol (TC), triglycerides (TGs), low-density lipoprotein cholesterol (LDL-C), and high-density lipoprotein cholesterol (HDL-C) levels to confirm the successful construction of the hypercholesterolemia model. Non-fasted blood samples were utilized at this intermediate stage to capture postprandial lipid peaks, which serve as highly sensitive indicators of dietary fat absorption and preliminary hypercholesterolemia establishment, thereby minimizing fasting-induced stress on the animals during the ongoing feeding protocol.

#### 2.6.2. Grouping and Intervention

After successful model construction, the hypercholesterolemic rats were randomly divided into four groups (15 rats per group): The Model Control group, and three experimental groups intervened by strain JS-51 with Lowdose, Mediumdose, or High dose. The intervention groups were fed with strain JS-51 suspensions (1.0 mL/100 g body weight) at concentrations of 1.0 × 10^6^ CFU/mL (Low dose), 1.0 × 10^7^ CFU/mL (Medium dose), and 1.0 × 10^8^ CFU/mL (High dose) by oral gavage, respectively (equivalent to administered doses of approximately 10^7^, 10^8^, and 10^9^ CFU/kg body weight per day, respectively). The Normal Control and Model Control groups were administered with an equivalent volume of sterile saline. During the 30-day intervention period, the Normal Control group was fed with the basal diet, while all other groups were fed with the high-fat diet.

#### 2.6.3. Determination of Blood Lipid Indices

At the conclusion of the 30-day intervention, all rats were fasted overnight. The animals were anaesthetized using isoflurane inhalation, and blood samples were collected via cardiac puncture. Following blood collection, all rats were euthanized by cervical dislocation to minimize suffering. Serum was then separated by centrifugation. The concentrations of serum total cholesterol (TC), triglycerides (TGs), high-density lipoprotein cholesterol (HDL-C), and low-density lipoprotein cholesterol (LDL-C) were quantitatively determined using commercial enzymatic kits on an automatic biochemical analyzer (BS-240VET, Mindray, Shenzhen, China).

#### 2.6.4. Determination of Visceral Indices

Body weight and food intake were recorded every 3 days throughout the experimental period to monitor the metabolic status of the rats. After sacrifice, the liver, heart, spleen, kidneys, and lungs of rats were excised, washed with saline, blotted dry, and weighed. The organ index was calculated using the following formula:

$$\mathrm{OrganIndex}(\%)=\frac{\mathrm{Organwetweight}(g)}{\mathrm{Finalbodyweight}(g)}\times 100$$

#### 2.6.5. Histopathological Observation of Liver

Fresh liver tissues were excised and immediately fixed in 10% neutral buffered formalin. The fixed tissues were dehydrated through a graded ethanol series, embedded in paraffin, and sectioned. The sections were stained with hematoxylin and eosin (H&E). Histopathological changes, including hepatocyte morphology and the extent of lipid droplet accumulation (steatosis), were examined and photographed under a light microscope.

### 2.7. Statistical Analysis

In vitro experiments were performed in independent biological triplicates. For the in vivo study, 15 animals were initially assigned to each experimental group, and all animals were included in the final analysis (*n* = 15 per group). Statistical analysis was performed using SPSS 26.0 software (IBM, Armonk, NY, USA). Prior to ANOVA, the normality of data distribution and the homogeneity of variances were confirmed using the ShapiroWilk test and Levene’s test, respectively. Statistical approaches were selected based on the experimental design: one-way analysis of variance (ANOVA) followed by Duncan’s multiple range test was used for single-factor end-point comparisons (e.g., final serum lipid levels and organ indices). For experiments involving multiple factors, such as strain and incubation time (e.g., time-course growth curves and cholesterol removal efficiency over time), a two-way ANOVA was applied. A value of *p* < 0.05 was considered statistically significant. A value of *p* < 0.05 was considered statistically significant. All figures were generated using Origin 2021 software (Origin Lab, Northampton, MA, USA).

## 3. Results

### 3.1. Screening Results of Cholesterol-Lowering Strains from Jiangshui

**Figure 1:**
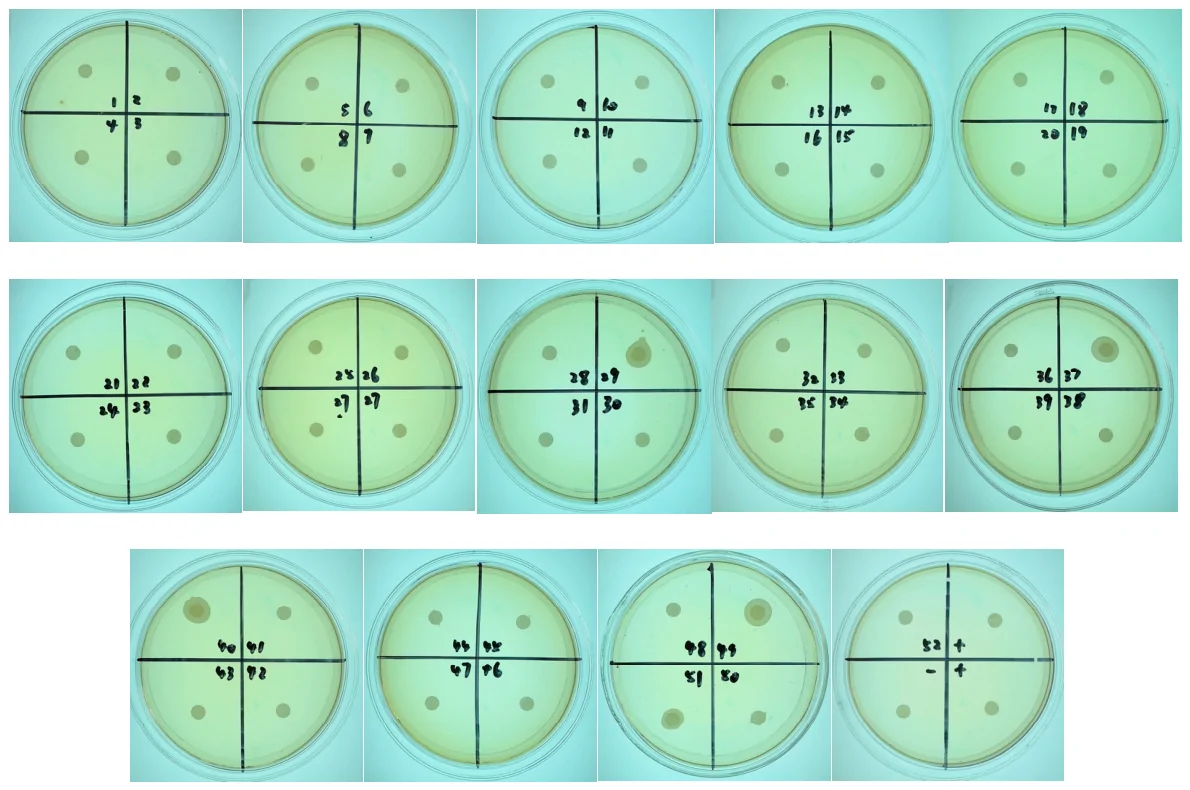
Culture results of isolated strains on the BSH solid medium. (The results of the preliminary isolation and purification of the strains are presented in the Supplementary Materials).

A total of 52 bacterial isolates exhibiting initial calcium dissolution halos were obtained from traditional fermented *Jiangshui* samples. Following isolation and purification via the streak-plate technique, macroscopic examination revealed that the majority of the colonies exhibited phenotypes characteristic of lactic acid bacteria [15]. The colonies appeared as creamy-white to yellowish opaque structures, characterized by convex surfaces and circular or irregular margins. These purified isolates were then subjected to confirmatory screening for BSH activity. In this assay, BSH-positive isolates were identified by the formation of precipitation zones, resulting from the interaction between hydrolyzed cholic acid and calcium carbonate. As shown in (Figure 1), there were obvious calcium precipitation zones around colonies of JS-29, JS-37, JS-40, JS-49, and JS-51. Based on the prominent hydrolysis and precipitation zones observed, these five promising strains were selected for further identification and physiological characterization.

### 3.2. Identification Results of Cholesterol-Lowering Strains from Jiangshui

**Figure 2:**
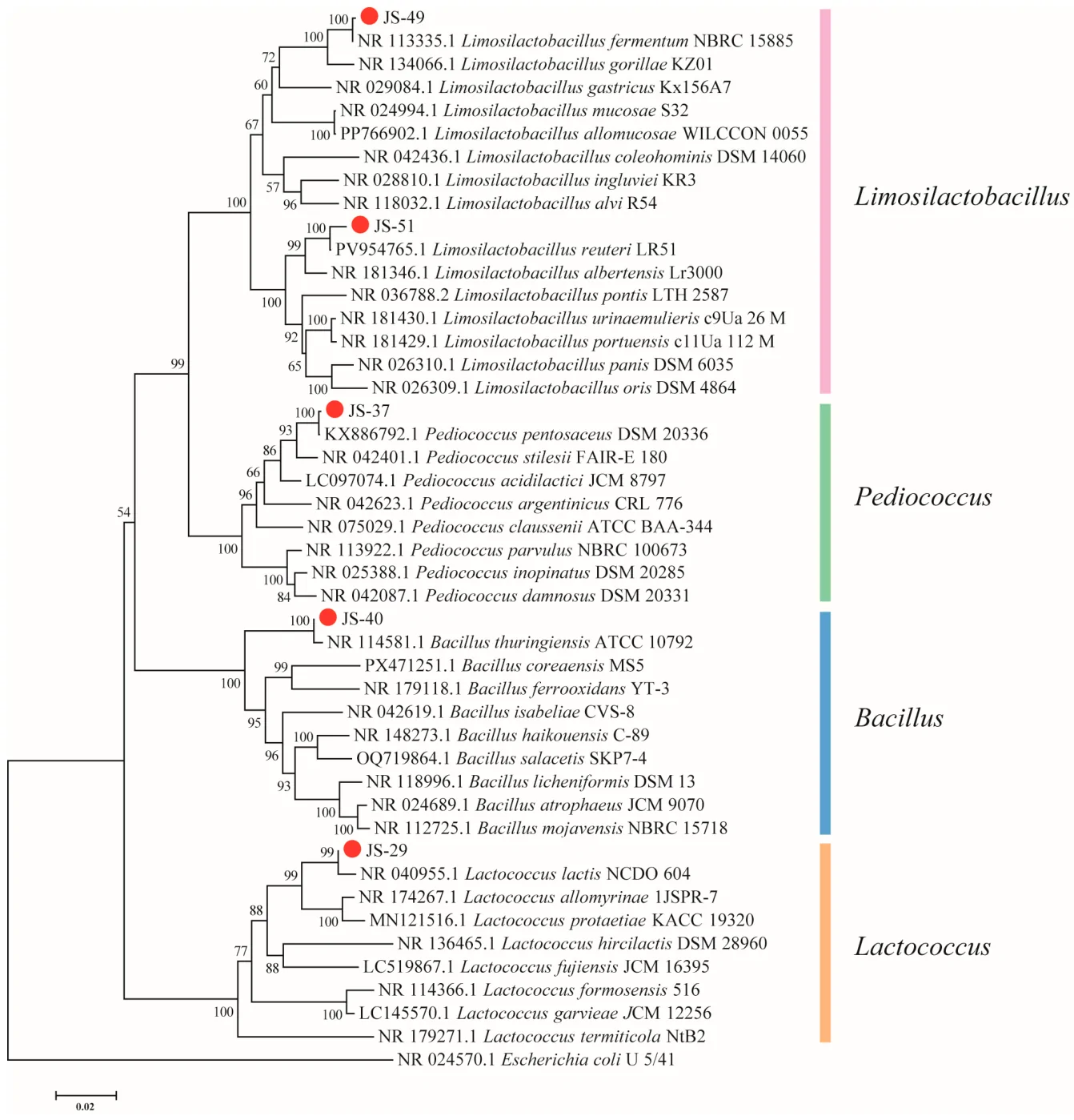
Phylogenetic trees of cholesterol-removing strains from Jiangshui.

**Figure 3:**
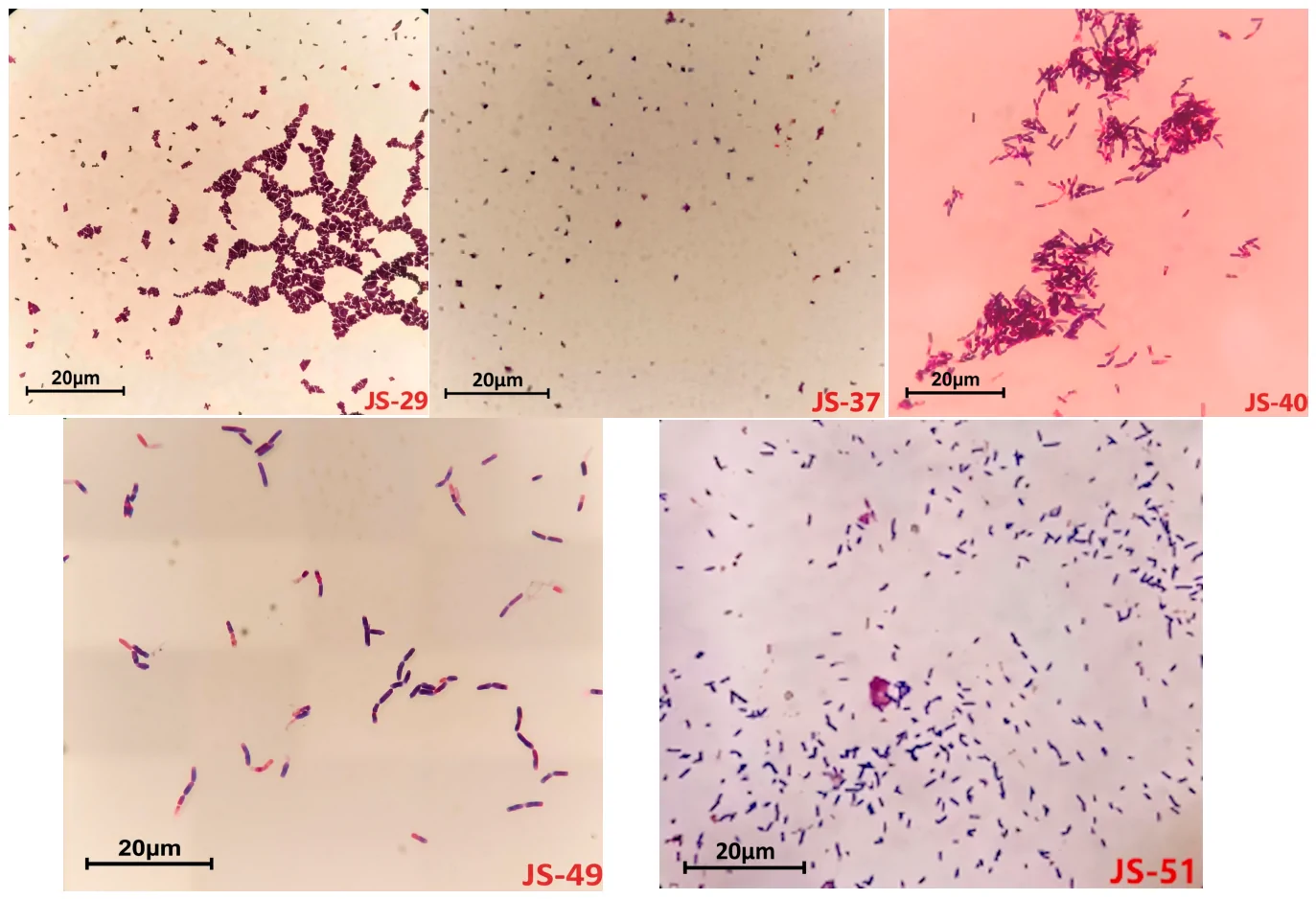
Gram-staining morphology of the five cholesterol-degrading strains (Scale bar = 20 μm).

The taxonomic identities of the five highly efficient isolates were definitively established via 16S rRNA gene sequencing coupled with phylogenetic analysis (Table 1 and Figure 2). Results of BLAST against the NCBI database confirmed that all sequences shared over 99.5% similarity with valid type strains. Strain JS-29 was identified as *Lactococcus lactis* (99.92% identity). Although it clustered within the *Lactococcus* lineage, the observed branch length discrepancy relative to the type strain *L. lactis* NCDO 604 is likely attributable to differences in the sequenced fragment length rather than true evolutionary divergence. Strains JS-49 and JS-51 were classified within the genus *Limosilactobacillus*, identified as *L. fermentum* (99.93%) and *L. reuteri* (99.86%), respectively. The isolation of *L. reuteri* is particularly significant given its extensive history of safe use as a functional candidate probiotic strain. Meanwhile, strain JS-40 clustered with *Bacillus thuringiensis* (100%). This result also confirmed that although *Bacillus* is taxonomically distinct from standard LAB, *Bacillus* species are common inhabitants of fermented foods and are well-documented to produce lactic acid and express bile salt hydrolase (BSH) activity [28]. The calcium dissolution halos around the colony of strain JS-40 observed during screening can likely be explained by this mechanism. Finally, strain JS-37 formed a strong monophyletic group with *Pediococcus pentosaceus* (99.93%). The results of microscopic observation showed that the cells of the five strains appeared purple after Gram staining (Figure 3), indicating that these five strains are Gram-positive bacteria. The results in Figure 3 show that strains JS-29 and JS-37 are cocci, while strains JS-40, JS-49, and JS-51 are rods. This phenotypic profile is consistent with the identification results of 16S rDNA sequencing. While these species are commonly associated with fermented foods, taxonomic assignment at the 16S rRNA level does not inherently establish strain-level safety, particularly for isolates like JS-40 (Bacillus thuringiensis). These identifications provided a preliminary biological rationale, but further rigorous strain-specific safety evaluations are necessary. The 16S rRNA gene sequences of the five isolated strains have been deposited in the NCBI GenBank database under the accession numbers QB052625–QB052629. Furthermore, to ensure full data transparency prior to their public release, the raw FASTA sequences are also provided in the Supplementary Materials.

### 3.3. Growth Performance of Cholesterol-Lowering Strains Under Different pH Conditions

**Figure 4:**
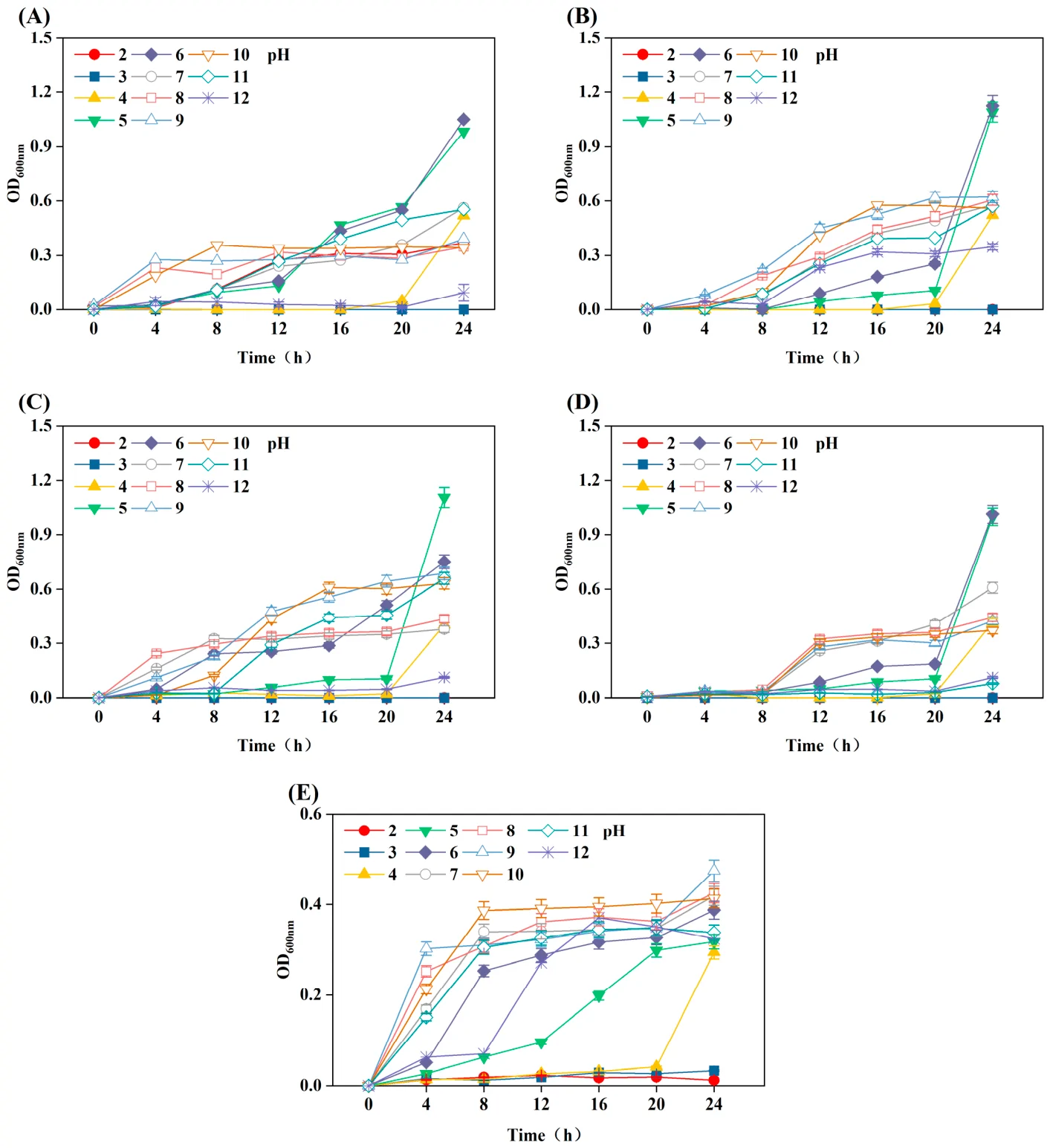
Growth curves of five cholesterol-degrading strains from Jiangshui under different pH environments. (**A**) Strain JS-29; (**B**) Strain JS-37; (**C**) Strain JS-40; (**D**) Strain JS-49; (**E**) Strain JS-51.

The growth ability of the 5 promising isolates under pH conditions ranging from 2.0 to 12.0 was quantitatively measured (Figure 4). Analysis of the time-dependent growth kinetics reveals that there is significant heterogeneity in the stress tolerance profiles of these strains. Upon exposure to acidic environments, specifically at pH ≤ 3.0, the proliferation of strains JS-29, JS-37, JS-40, and JS-49 was markedly inhibited, as their optical density (OD_600_) values showed almost no increase during the 24h incubation period. Conversely, strain JS-51 exhibited slightly better growth capability under acidic environments. In terms of tolerance to alkaline environments, most strains can hardly proliferate when the pH value reaches 12.0, whereas strains JS-37 and JS-51 exhibited good proliferation capabilities. Notably, JS-51 not only survived but continued to accumulate biomass at this extreme alkalinity. However, as OD_600_ measurements can be influenced by medium precipitation at high pH, further verification via viable cell counts (CFU) is warranted.

### 3.4. Viability of 5 Cholesterol-Lowering Strains in Low pH Environments

**Figure 5:**
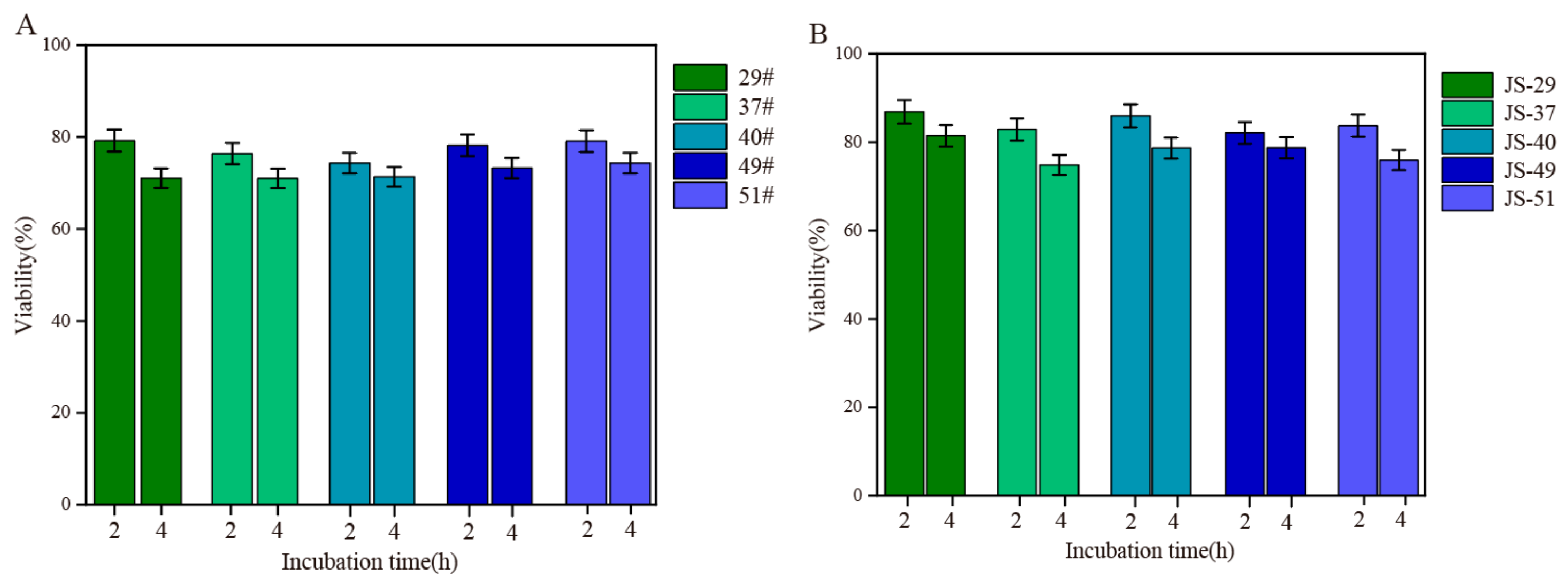
Survival rate of five cholesterol-degrading strains under low pH conditions. (**A**) Survival rate under pH 2.0; (**B**) urvival rate under pH 3.0.

To evaluate the tolerance of the 5 cholesterol-lowering strains to gastric acid, the survival ability of the five strains was measured under conditions of pH 2.0 (Figure 5A) and pH 3.0 (Figure 5B). The results indicated that all 5 strains exhibited good stability in highly acidic environments. After incubation for 2 and 4 h at these pH environments, the survival rate of all strains remained above 70%. Only a marginal decline in survival rate over time was observed, indicating that these strains possess the ability to resist harsh acidic conditions.

### 3.5. Growth Performance of 5 Cholesterol-Lowering Strains from Jiangshui in Bile Salt Environments

**Figure 6:**
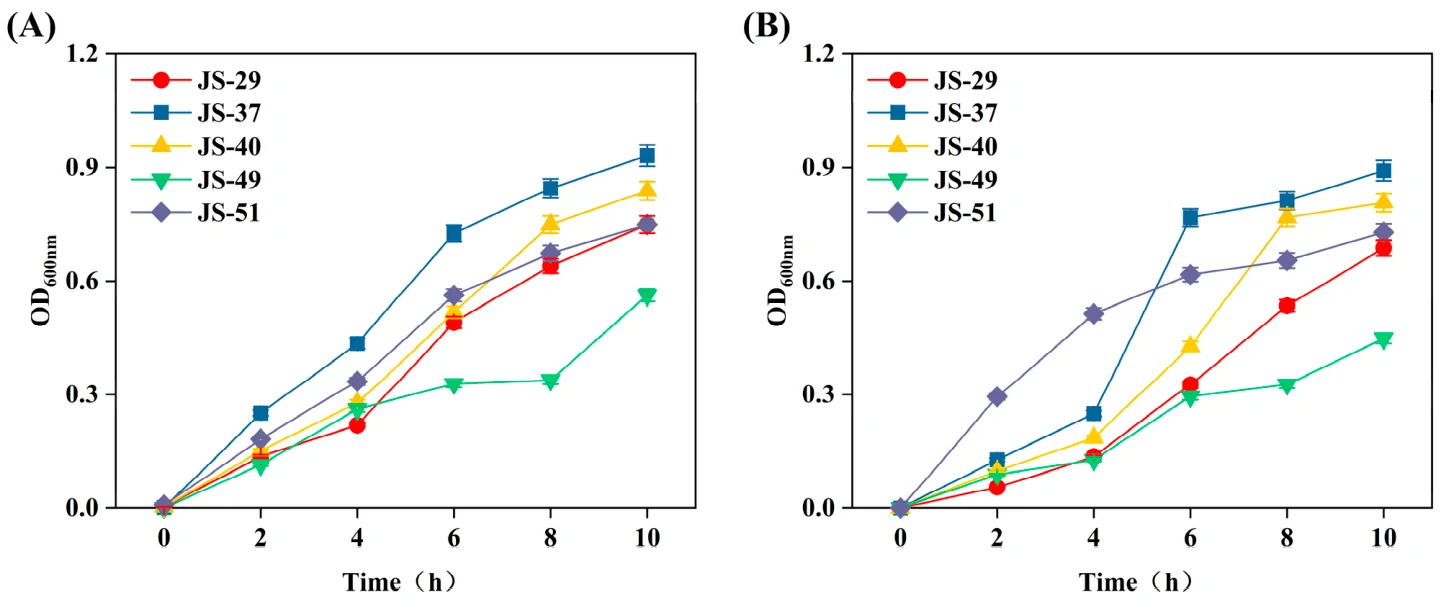
Growth ability of five cholesterol-degrading strains in different Bile Salt Concentrations. (**A**) Bile salt concentration: 0.3%; (**B**) Bile salt concentration: 0.5%.

The proliferative capacity of the five isolates was monitored over a 10h incubation period in MRS broth supplemented with 0.5% (*w*/*v*) bile salts (Figure 6). The results of time-dependent growth kinetics revealed that there is substantial variability among these strains in response to bile stress. For strains JS-29 and JS-49, the high concentration of bile salts imposed a severe bacteriostatic effect, characterized by a flattened growth curve with a prolonged lag phase and minimal biomass accumulation; their final optical density (OD_600_) values remained comparatively low. In distinct contrast, strains JS-37, JS-40, and JS-51 exhibited stronger growth capabilities, indicating their good tolerance to bile salts. In the medium with a bile salt concentration of 0.5%, the growth rates of strains JS-37 and JS-40 were relatively slow from 0 to 4 h, but their growth rates significantly increased from 4 to 8 h, while the strain JS-51 maintained a relatively high growth rate from 0 to 8 h. After being cultured for 10 h, the OD_600_ values of JS-37, JS-40, and JS-51 all exceeded 0.7, indicating that they were able to recover from initial stress and resume culture turbidity increase and biomass accumulation. Among them, JS-37 achieved the highest final density, closely followed by JS-40 and JS-51. These data clearly delineate these isolates into bile-sensitive and bile-tolerant phenotypes, with the latter group effectively maintaining biomass accumulation despite the rigorous 0.5% bile salt challenge.

### 3.6. Measurement Results of Strain Hydrophobicity

**Figure 7:**
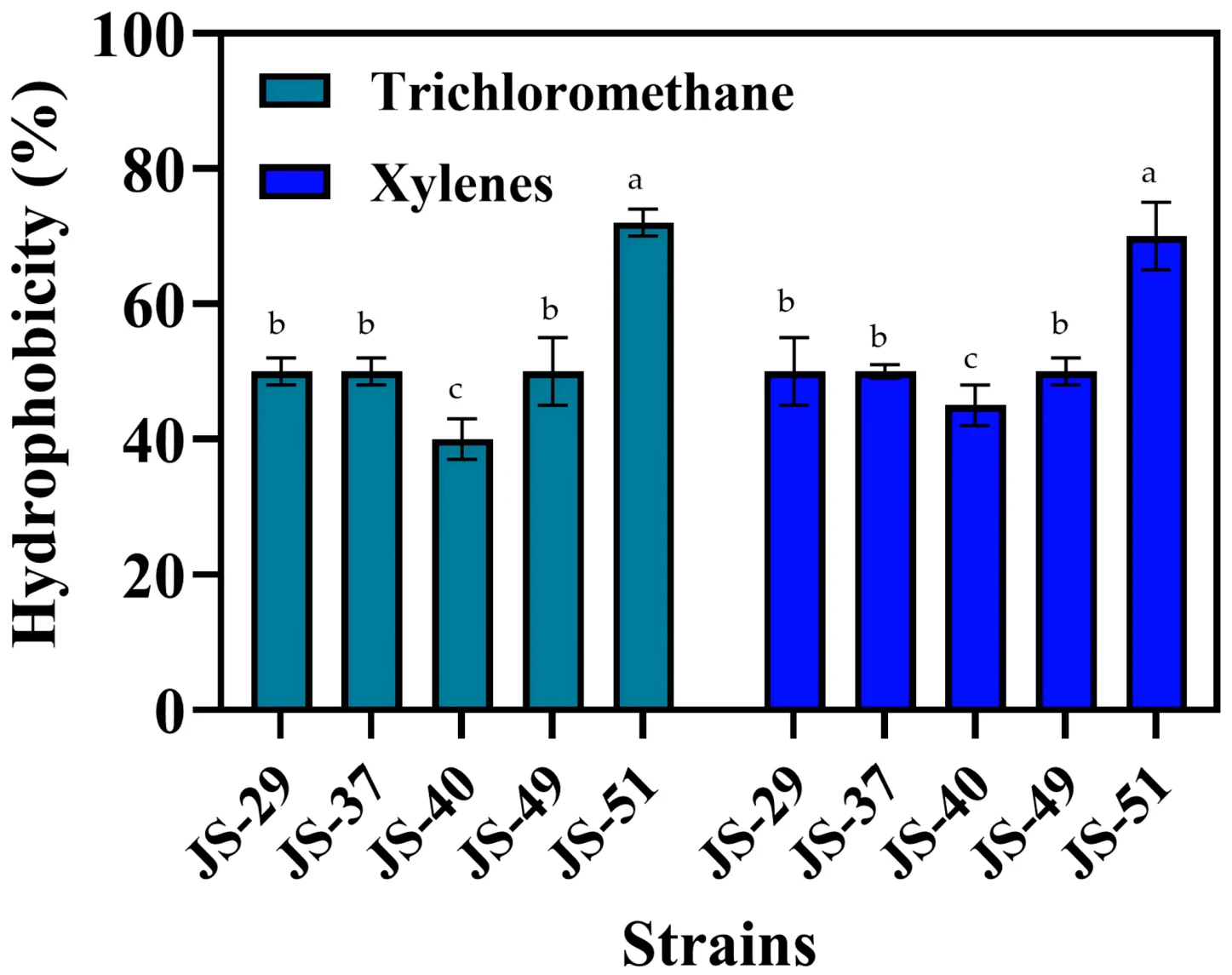
Hydrophobicity of five cholesterol-degrading strains. Data are expressed as mean ± SD (*n* = 3). Different lowercase letters (a, b, c) indicate statistically significant differences between strains (*p* < 0.05).

The cell surface hydrophobicity (CSH) of the five candidate strains was quantitatively characterized using the microbial adhesion to hydrocarbons (MATH) assay (Figure 7). Statistical analysis of the adhesion data revealed significant heterogeneity in the surface physicochemical properties among the tested isolates (*p* < 0.05). A distinct stratification in hydrophobic capabilities was observed. Strains JS-29, JS-37, JS-40, and JS-49 exhibited a moderate hydrophobic phenotype, with an adhesion percentage not exceeding 51%, while strain JS-51 demonstrated a significantly stronger affinity for organic solvents. The highest hydrophobicity index of strain JS-51 achieved 72.36 ± 1.24% [29]. As indicated by the statistical significance markers (superscript letters) in Figure 7, the performance of strain JS-51 was statistically distinct from all other candidates. Although the other isolates showed comparable intermediate values, strain JS-51 surpassed the second-highest strain by a significant margin. This data identifies strain JS-51 as the unique outlier with the most pronounced surface non-polarity in the screened library, exhibiting an adhesive ability substantially surpassing the average level of the group.

### 3.7. Kinetics of Cholesterol Removal by 5 Strains In Vitro

**Figure 8:**
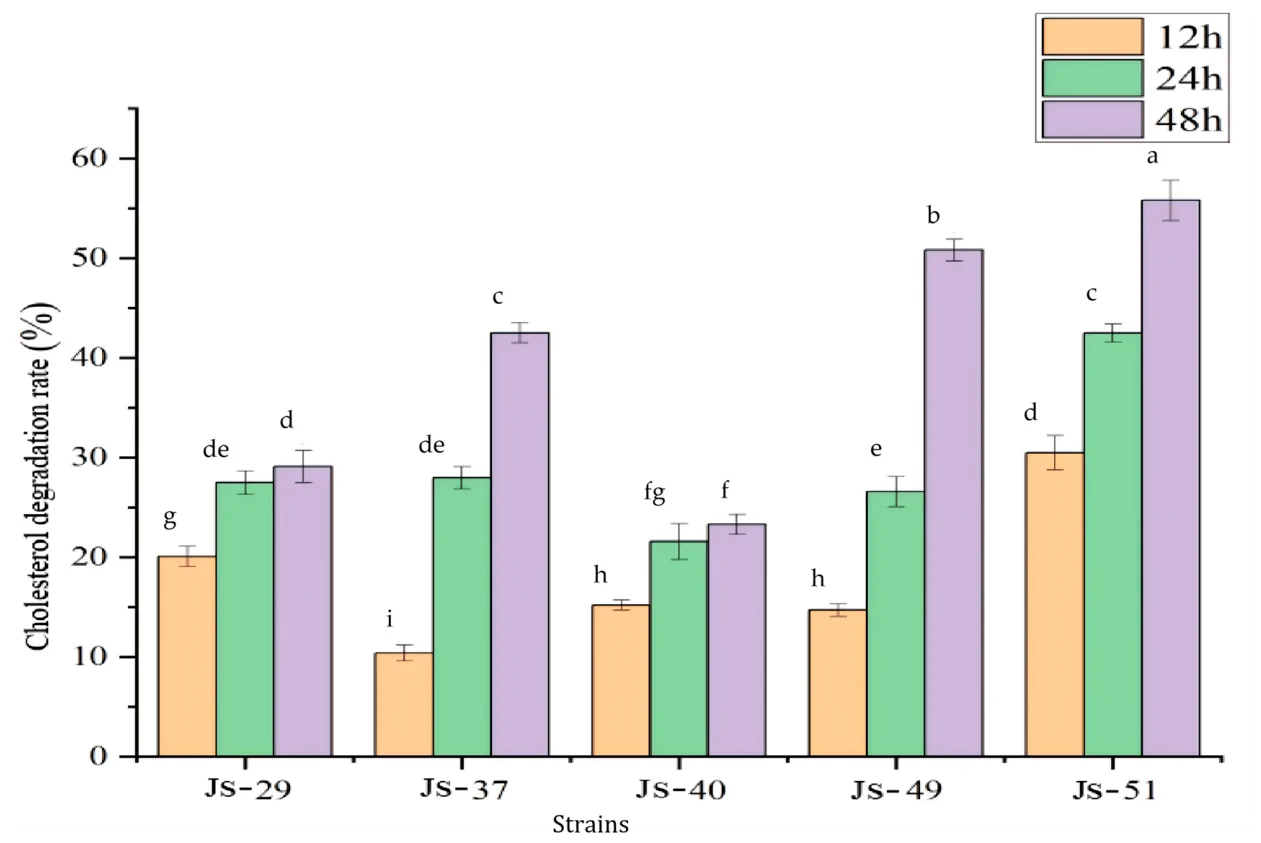
Cholesterol-removal rates of the five strains in vitro. Data are expressed as mean ± SD of three independent experiments (*n* = 3). Different lowercase letters indicate statistically significant differences (*p* < 0.05).

The cholesterol removal efficiencies of the five isolates were monitored over a 48h incubation period to determine their kinetic profiles (Figure 8). The reaction solutions of all strains exhibited a time-dependent reduction in cholesterol concentrations. However, significant differences were observed among the strains (*p* < 0.05). Strain JS-51 consistently demonstrated the highest removal capacity at all measured intervals. During the early phase (12 h), JS-51 achieved a removal rate of 30.21%, establishing an immediate statistical lead over JS-29 (20.06%). At 24 h, the cholesterol removal rate of JS-51 had increased to 41.86%, significantly surpassing the intermediate performance of JS-29 and JS-37, whose removal rates were around 28%. At the end of the 48h experiment, the maximum cholesterol removal rate of JS-51 reached its peak, which was 55.15 ± 1.87%. Statistical analysis confirmed this value as the highest in the screened library, significantly exceeding that of the second-best strain JS-49 (51.27%). Notably, while strains such as JS-40 and JS-29 exhibited a kinetic plateau between 24 and 48 h, JS-51 maintained a steep, continuous upward trajectory. This data identifies JS-51 as the most efficient candidate, characterized by a sustained and non-saturating removal capability throughout the experimental duration.

### 3.8. Initial Body Weight Analysis Results of Rats

Following the adaptive feeding period, the 75 rats were randomly assigned to the Normal Control group (n = 15) and the Model group (n = 60). The initial body weights for both groups are presented in Table 2. The mean body weight of the Normal Control group was 196.5 g, while that of the Model group was 195.9 g. Statistical analysis indicated no significant difference in the initial body weights between the two groups (*p* > 0.05).

### 3.9. Construction Results of High-Fat Rat Model

**Figure 9:**
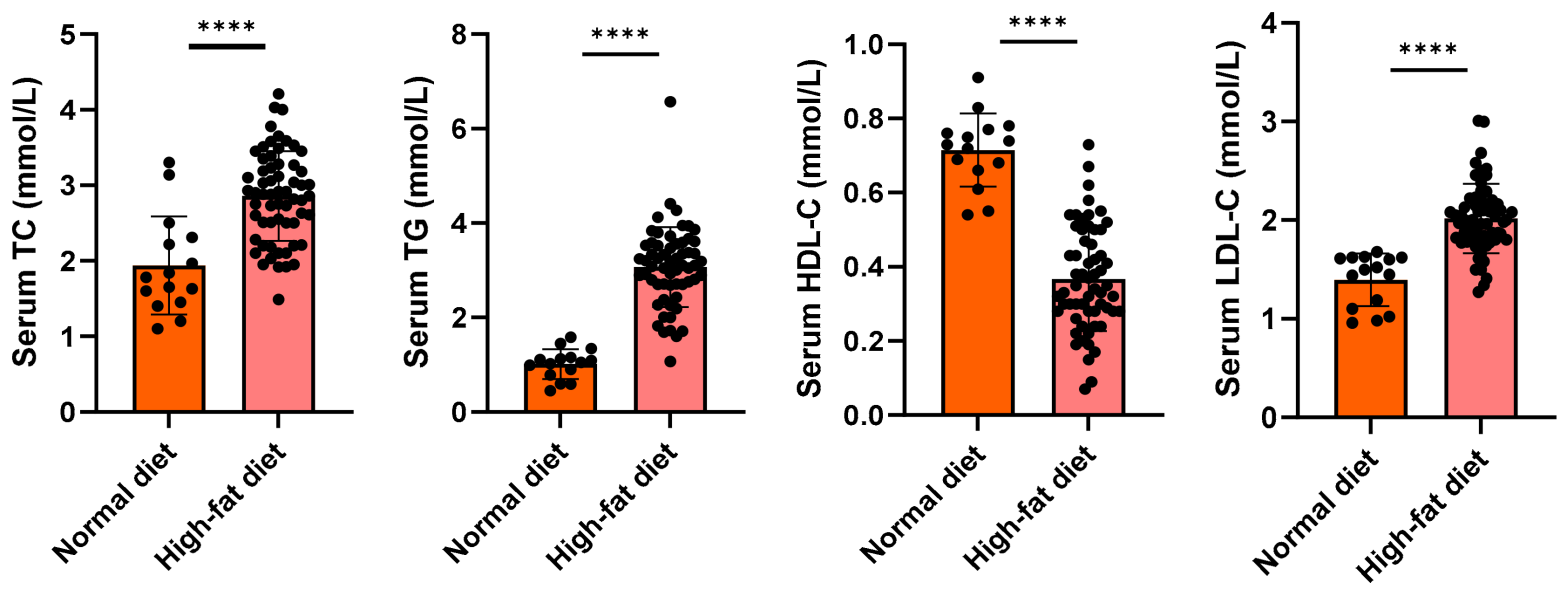
Blood lipid levels of rats in each group after modeling. Black dots represent individual data points. **** indicates a highly significant difference compared to the Normal diet group (*p* < 0.0001).

After four weeks of continuous feeding with the high-fat diet, to assess whether the high-fat model was successfully established, non-fasted blood from 75 rats was collected. Following blood collection, serum was separated and the levels of serum total cholesterol (TC), triglycerides (TGs), low-density lipoprotein cholesterol (LDL-C), and high-density lipoprotein cholesterol (HDL-C) were measured. The quantitative data and comparative trends are presented in Table 3 and Figure 9, respectively. As shown in Table 3, the HFD significantly altered the lipid profile of rats. Compared to the Normal Control group maintained on a basal diet, the rats in the HFD group exhibited markedly elevated levels of serum TC, TG, and LDL-C (*p* < 0.0001). Simultaneously, a significant decrease in serum HDL-C levels was observed in the rats of the HFD group (*p* < 0.0001, Figure 9). This specific dyslipidemic pattern, characterized by the accumulation of pro-atherogenic lipids and the depletion of cardioprotective lipoproteins, is consistent with the classical physiological manifestations of diet-induced hyperlipidemia described in previous studies. These results not only confirm that the hyperlipidemia rat model has been successfully constructed, but also validate the grouping strategy for subsequent candidate probiotic strain interventions.

### 3.10. The Effect of Strain JS-51on Blood Lipids in Hyperlipidemia Rats

**Figure 10:**
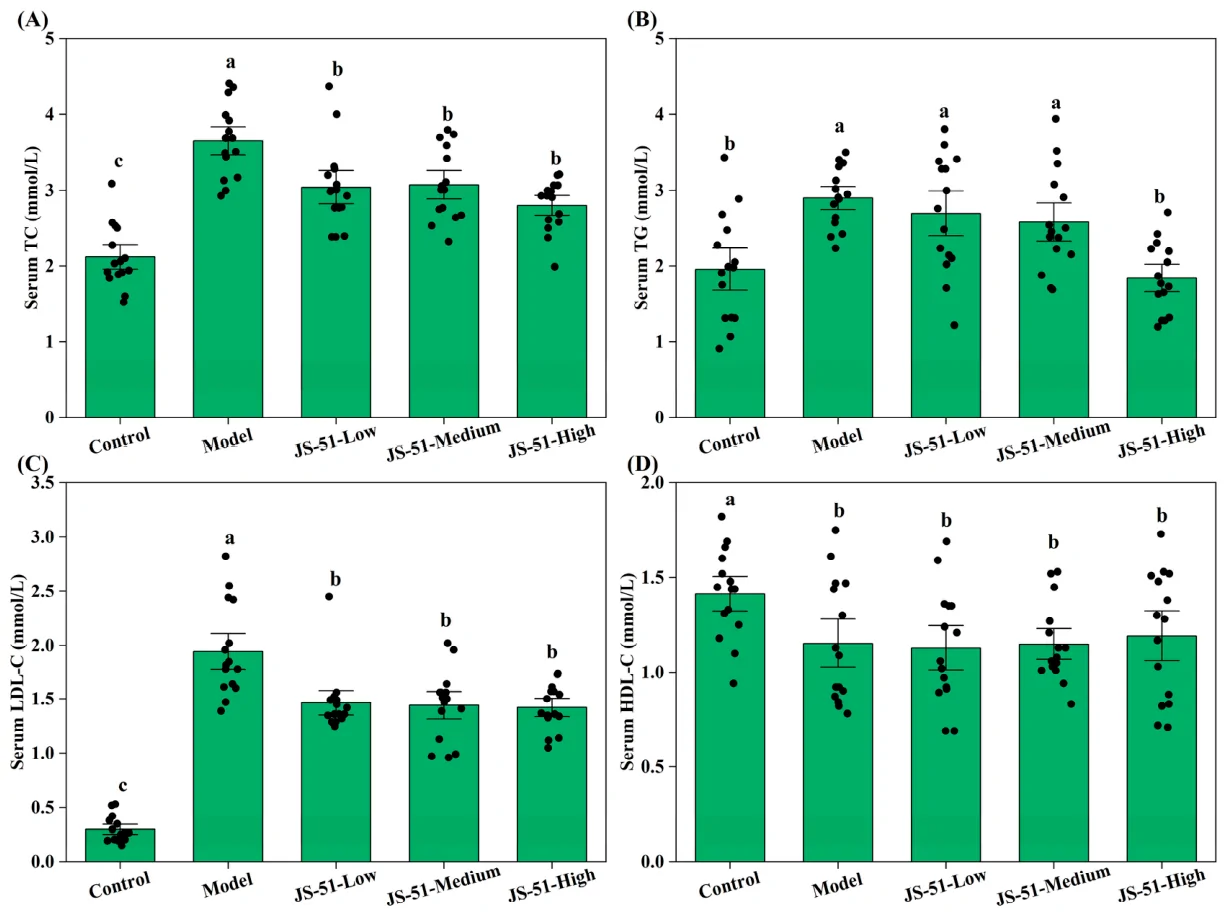
The effect of strain JS-51 on blood lipid levels in rats. (**A**) Serum total cholesterol (TC); (**B**) Serum triglycerides (TG); (**C**) Serum low-density lipoprotein cholesterol (LDL-C); (**D**) Serum high-density lipoprotein cholesterol (HDL-C). Data are presented as mean ± SD (*n* = 15 per group). Black dots represent individual data points. Different lowercase letters (a, b, c) indicate statistically significant differences between groups (*p* < 0.05).

Following the 30-day experimental period, fasted serum lipid profiles were quantified to assess the intervention effects at the experimental endpoint (Figure 10). Since intermediate lipid measurements (prior to intervention) were taken in a non-fasted state to confirm model establishment, direct longitudinal comparisons were confounded by the differing fasting status. Therefore, the therapeutic efficacy was statistically evaluated based solely on cross-sectional between-group differences at the final endpoint using one-way ANOVA.

### 3.11. The Effect of Strain JS-51on the Body Weight of Hyperlipidemia Rats

**Figure 11:**
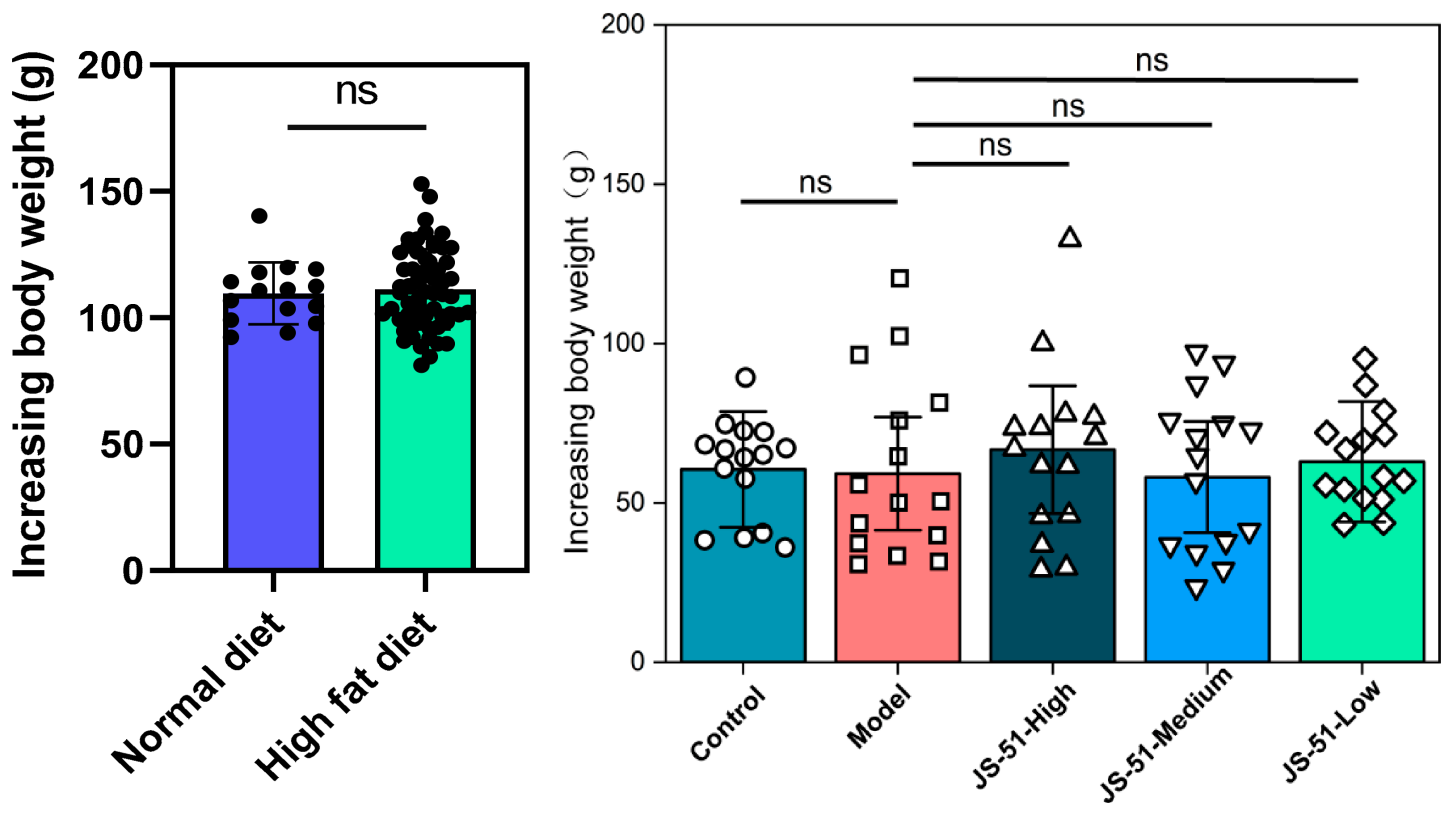
Weight gain of rats after modeling and treatment. (**Left**) The weight gain of rats after modeling. (**Right**) The weight gain after administration. Data are presented as mean ± SD (*n* = 15 per group). Symbols (e.g., dots, squares, triangles) represent individual data points. "ns" indicates no significant difference (*p* > 0.05).

The weight gain of rats after modeling and intervention treatment is shown in Figure 11. The left panel displays the weight gain of rats in the normal diet group and the high-fat diet group after four weeks of feeding, while the right panel presents the body weight increase after administration in the Control, Model, and JS-51 intervention groups (51-High, 51-Medium, and 51-Low dosages). The results indicated no significant difference in weight gain between the Normal Diet and High-Fat Diet groups (*p* > 0.05, marked as “ns”), suggesting that the high-fat diet did not induce significant weight changes under the experimental conditions. Furthermore, supplementation with different doses of JS-51 did not demonstrate a statistically significant impact on body weight gain. Regardless of the dosage (High, Medium, or Low), no significant differences were observed in weight increments compared to the Model and Control groups. These data suggest that the intervention with strain JS-51 had a limited effect on overall weight regulation in this study, which may be attributed to the specific animal model or the relatively short intervention duration. Additionally, no statistically significant differences in daily average food consumption were observed among the Model and JS-51 intervention groups throughout the experimental period (*p* > 0.05), indicating that the observed effects were independent of caloric intake.

### 3.12. The Influence of Strain JS-51 on Organ Coefficients in Hyperlipidemia Rats

**Figure 12:**
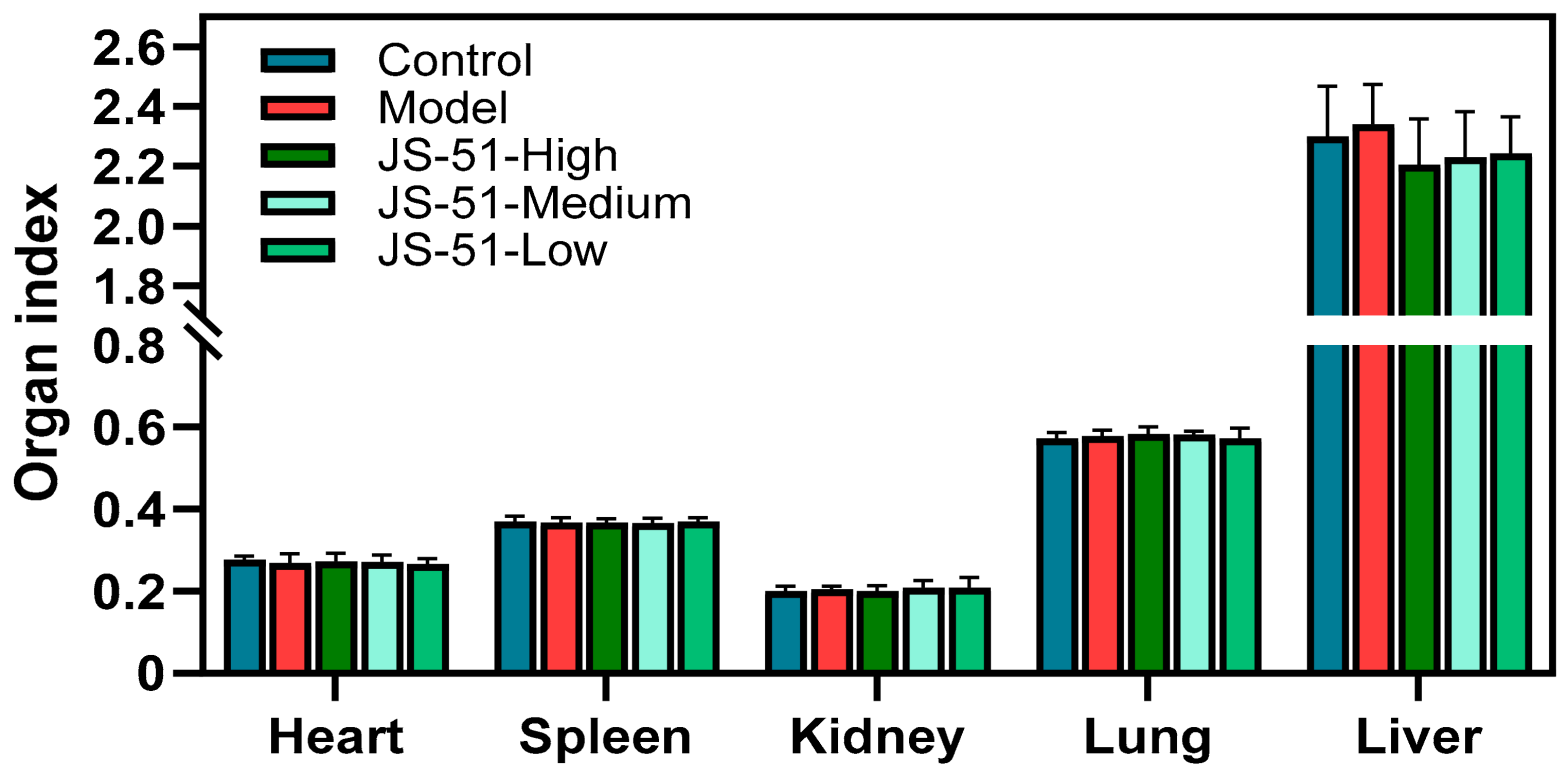
The effect of strain JS-51 on organ coefficients in rats. Data are presented as mean ± SD (n = 15 per group).

Post-mortem gravimetric analysis was conducted to determine organ coefficients, defined as the ratio of organ wet weight to final body weight, for the heart, spleen, kidneys, and liver (Figure 12). This metric serves as a macroscopic indicator of potential anatomical alterations. For the non-target organs, especially the heart, spleen, and kidneys, the data indicate that all experimental groups exhibited high physiological stability in these organs. Statistical evaluation confirmed that there were no significant inter-group differences in the indices of these visceral organs between strain JS-51 intervention groups and the Control group (*p* > 0.05), suggesting that the treatment did not induce macroscopic hypertrophy or atrophy in these tissues. In distinct contrast, the hepatic coefficient exhibited significant variation in response to the high-fat diet and candidate probiotic strain interventions. The Model group displayed a pronounced elevation in liver index, reaching 2.34 ± 0.13%, which was statistically higher than that of the Control group (*p* < 0.05). This increase quantitatively reflects the hepatic enlargement induced by a high-fat diet. However, the oral administration of strain JS-51 effectively modulated this parameter. A dose-dependent downward trend was observed, with the high-dose intervention (1.0 × 10^8^ CFU/mL) exerting the most significant impact. The liver index in the high-dose group was reduced to 2.21 ± 0.15%, representing a quantitative decrease of 5.56% relative to the untreated Model group. This result indicates that the increase in liver mass induced by a high-fat diet can be reversed by strain JS-51.

### 3.13. The Impact of Strain JS-51 on the Liver Structure of Hyperlipidemia Rats

**Figure 13:**
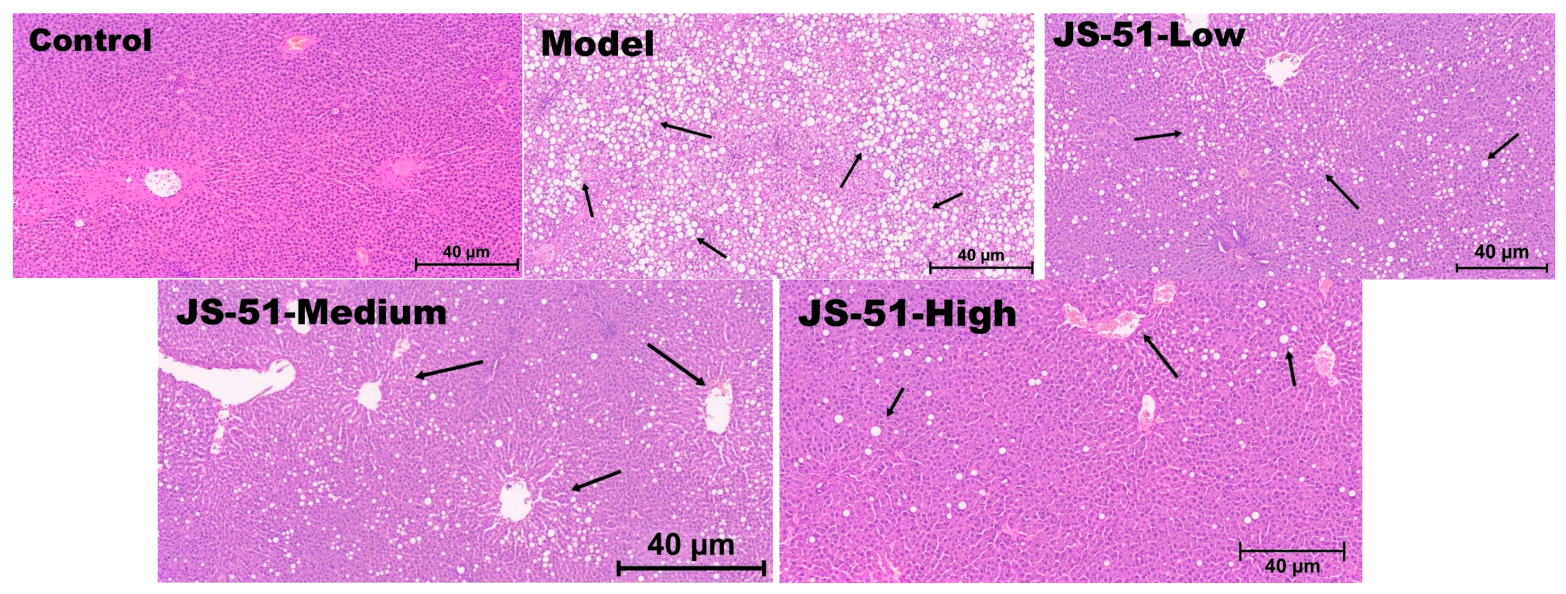
Microscopic observation images of rat liver slices. (Scale bar = 40 μm). Black arrows indicate the unstained clear spaces (lipid vacuoles) representing hepatic steatosis.

The histopathological alterations in hepatic tissue were evaluated using hematoxylin-eosin (H&E) staining (Figure 13). Microscopic examination of the Control group revealed a normal hepatic architecture, characterized by radially arranged hepatic cords and hepatocytes with distinct polygonal morphology, uniform eosinophilic cytoplasm, and centrally located nuclei. No evidence of pathological lipid accumulation was observed. Conversely, the liver sections from the Model group exhibited typical severe morphological deviations of steatosis. The cytoplasm was heavily occupied by diffuse lipid vacuoles of varying sizes (micro- and macrovesicular steatosis), leading to widespread hepatocyte ballooning [30]. Consequently, this excessive accumulation of intracellular lipids resulted in the displacement of nuclei toward the cell periphery, exhibiting distinct nuclear marginalization. Following administration, strain JS-51 induced a visible, dose-related improvement in hepatic morphological structure. While the low-dose and medium-dose groups showed intermediate levels of clearance, the high-dose group (1.0 × 10^8^ CFU/mL) demonstrated the most profound histological recovery. In the high-dose experimental group, the density and size of lipid vacuoles were drastically reduced. The hepatocytes largely regained their structural integrity, with nuclei returning to their physiological central position. The overall histological phenotype of the high-dose group was comparable to that of the Control group, suggesting a qualitative reduction in lipid vacuole density relative to the untreated Model group. It should be noted that because the liver samples were paraffin-embedded, intracellular lipids were inherently extracted during tissue processing, leaving unstained clear spaces (vacuoles) in the hepatocytes. The density and size of these macro- and microvesicular clear spaces were utilized as standard histopathological surrogate markers for evaluating steatosis severity.

## 4. Discussion

As hypercholesterolemia is a primary risk factor for ASCVD, finding safe, functional-food based strategies for managing lipid levels is essential [1]. While pharmacological interventions such as statins are effective, the growing consumer demand for natural alternatives has positioned functional fermented foods as a promising frontier for long-term cardiovascular health management. Unlike conventional candidate strains often sourced from dairy matrices or general gut isolates, the present study focused on *Jiangshui*, a traditional fermented vegetable beverage from Northwest China characterized by a distinctively acidic ecological niche (pH 3.2–3.5) [14]. We hypothesized that this harsh fermentation environment imposes a rigorous selective pressure, favoring the evolution of lactic acid bacteria (LAB) with superior stress tolerance and strong colonization potential. The isolation and characterization of *Limosilactobacillus reuteri* JS-51 in this study suggest that this specific batch of indigenous ethnic food serves as a potential source capable of surviving the hostile conditions of the mammalian gastrointestinal tract.

The cholesterol-lowering efficacy of *L. reuteri* JS-51 is likely mediated by a synergistic mechanism involving both enzymatic bile salt hydrolase (BSH) activity and physical cell surface properties. BSH activity is widely regarded as the primary metabolic pathway for cholesterol reduction [31,32]. BSH-active strains facilitate the precipitation and fecal excretion of bile acids by hydrolyzing conjugated bile salts into deconjugated bile acids. This disruption of the enterohepatic circulation compels the liver to recruit elevated levels of endogenous cholesterol for the *de novo* synthesis of bile acids, thereby lowering serum cholesterol pools [33]. In vitro assays demonstrated that strain JS-51 possesses strong BSH activity, evidenced by prominent calcium precipitation rings and a substantial cholesterol removal efficiency of 55.15% over 48 h. Crucially, this enzymatic mechanism is complemented by the exceptional cell surface hydrophobicity (72.36%) of strain JS-51, a trait significantly higher than typical values reported for generic *Lactobacillus* species. High hydrophobicity is physically correlated with the presence of specific cell wall proteins or exopolysaccharides, such as S-layer proteins [29], that facilitate the non-specific sequestration and co-precipitation of cholesterol molecules [22,34]. Moreover, this surface property is a key determinant of intestinal adhesion. The superior hydrophobicity of strain JS-51 suggests a potential capability to adhere to the intestinal epithelium, thereby prolonging its residence time and maximizing its metabolic interaction with dietary lipids.

Physiological robustness is a prerequisite for these mechanisms to function in vivo. A standout feature of strain JS-51 is its vigorous growth at pH 3.0, a phenotype likely acquired through environmental domestication in the acidic *Jiangshui* matrix [14,15,16,17,18]. This intrinsic acid resilience, combined with its tolerance to high concentrations of bile salts (0.5%), may facilitate the survival of a sufficient viable load of strain JS-51 passing through the gastric acid barrier and enter the small intestine for colonization. The therapeutic effects of these traits in vitro were substantiated in the high-fat diet (HFD)-induced hypercholesterolemic rat model. Oral administration of strain JS-51 elicited a profound, dose-dependent amelioration of dyslipidemia, characterized by significant reductions in serum TC, TG, and LDL-C, alongside a concurrent elevation of cardioprotective HDL-C [8,9]. Beyond serum parameters, the histological attenuation of hepatic steatosis—evidenced by the clearance of lipid droplets and restoration of hepatocyte morphology—suggests a potential beneficial interaction along the gutliver axis [35,36]. We hypothesize that this might be mediated by enhanced bile acid deconjugation, which could subsequently promote fecal bile acid excretion and modulate hepatic LDL receptor expression [37,38], though these specific molecular pathways and in vivo bile acid profiles were not directly quantified in the present study. Thus, strain JS-51 effectively alleviates the hepatic lipid burden and restores metabolic homeostasis. Interestingly, while serum lipid profiles were significantly improved, the supplementation of strain JS-51 did not alter body weight gain, which differs from some anti-obesity strains [39]. This aligns with the findings of [40], suggesting that certain candidate strains may decouple metabolic regulation from weight loss. This implies that *L. reuteri* JS-51 specifically targets lipid metabolism pathways [41,42] (e.g., bile acid deconjugation via BSH) and hepatic lipid export, rather than influencing energy intake [43] or absorption. This specific trait suggests *L. reuteri* JS-51 could be particularly suitable for managing dyslipidemia in non-obese individuals or as a candidate for further investigation in dietary interventions when weight maintenance is desired. Collectively, these findings position *L. reuteri* JS-51 as a potent, naturally evolved probiotic candidate, offering a viable functional food strategy.

Limitations: Despite the promising phenotypic outcomes, several methodological limitations must be acknowledged. Firstly, taxonomic identification relies on 16S rRNA gene sequencing. Definitive strain-level classification and comprehensive safety profiling—including hemolytic activity, phenotypic antibiotic susceptibility, and the absence of transferable antimicrobial-resistance and virulence-associated genes (especially for JS-40)—require future whole-genome sequencing (WGS) prior to dietary applications. Secondly, cellular survival under extreme stress (pH 12 and 0.5% bile salts) was evaluated via optical density; future validation employing direct CFU enumeration is necessary. Thirdly, the mechanisms underlying cholesterol removal were deduced from in vitro phenotypic assays; multi-omics data, gut microbiota profiling, and quantitative measurements of fecal bile acids are needed to conclusively prove these gutliver axis pathways in vivo. Lastly, the histological amelioration of hepatic steatosis was evaluated primarily through qualitative H&E imaging of paraffin sections; future studies should incorporate lipid-specific staining (e.g., Oil Red O on frozen sections) and blinded quantitative image analysis to substantiate absolute lipid accumulation. Ultimately, further rigorous clinical trials are imperative to validate the safety, optimal dosage, and long-term lipid-lowering efficacy of this strain in human populations.

## 5. Conclusions

This study validates the efficacy of an ecological screening strategy targeting traditional fermented foods for identifying robust probiotic candidates [11]. We successfully identified *Limosilactobacillus reuteri* JS-51 from *Jiangshui*, a distinctively acidic vegetable ferment from Northwest China. This strain distinguishes itself through exceptional resilience to low pH (pH 3.0) and high concentrations of bile salts, traits likely acquired through environmental domestication. Based on our in vitro findings, we hypothesize that the cholesterol-lowering potential of *L. reuteri* JS-51 may involve a synergistic action, combining potent bile salt hydrolase (BSH) activity with high-affinity cell surface hydrophobicity, ensuring both enzymatic deconjugation and physical sequestration of luminal lipids. In the high-fat diet-induced hypercholesterolemic rat model, oral administration of *L. reuteri* JS-51 significantly improved dyslipidemia and alleviated hepatic steatosis [44], contributing to the morphological improvement of the liver architecture. These findings strongly suggest that *L. reuteri* JS-51 exerts a hepatoprotective effect, potentially via the modulation of bile acid metabolism within the gut-liver axis, thereby mitigating systemic lipotoxicity [30,45]. Based on these results, *L. reuteri* JS-51 has strong potential as a functional starter culture for the development of bioactive fermented foods. Future investigations should focus on validating the structural changes in gut microbiota via metagenomic sequencing, elucidating the molecular signaling pathways (e.g., FXR-FGF15 axis) [46], and conducting clinical trials to fully translate these preclinical benefits into human cardiovascular health management [47,48].

## Figures and Tables

**Table 1:** 16S rDNA sequence alignment results.

Strain Number	Strain with Highest Homology	Species Name	Identity Percentage
JS-29	*Lactococcus lactis NCDO 604*	*Lactococcus lactis*	99.92%
JS-37	*Pediococcus pentosaceus DSM 20336*	*Pediococcus pentosaceus*	99.93%
JS-40	*Bacillus thuringiensis ATCC 10792*	*Bacillus thuringiensis.*	100%
JS-49	*Limosilactobacillus fermentum NBRC 15885*	*Limosilactobacillus fermentum*	99.93%
JS-51	*Limosilactobacillus reuteri LR51*	*Limosilactobacillus reuteri*	99.86%

**Table 2:** Initial body weight of the blank control group and model group before modeling.

Group	Number of Animals (Individuals)	Initial Body Weight (g)
Blank control group	15	196. 1± 6.7
Model group	60	195.9 ± 6.4

**Table 3:** Lipid levels of rats in each group after modeling.

Index	Normal Diet	High Fat Diet	*p*-Value
TC (mmol/L)	1.87 ± 0.56	2.89 ± 0.57	<0.0001
TG (mmol/L)	1.02 ± 0.32	3.07 ± 0.85	<0.0001
HDL-C (mmol/L)	0.71 ± 0.09	0.37 ± 0. 14	<0.0001
LDL-C (mmol/L)	1.39 ± 0.27	2.02 ± 0.35	<0.0001

## Data Availability

The original 16S rRNA sequence data presented in the study are included in the article’s Supplementary Materials and have been submitted to the NCBI GenBank database under accession numbers QB052625–QB052629.

## Supplementary Materials

The following supporting information can be downloaded at: https://www.mdpi.com/article/10.3390/foods15193554/s1, Figure S1: Strain isolation by dilution; Figure S2: Colony morphology of the strain (-1); Figure S3: Colony morphology of the strain (-2); Data S1: 16S rRNA gene sequences of the isolated strains.
